# Circulating METRNL, Adipocytokines, and Insulin-Resistance Markers After Repeated Whole-Body Cryotherapy in Women of Different Ages

**DOI:** 10.3390/ijms27136019

**Published:** 2026-07-04

**Authors:** Magdalena Wiecek, Malgorzata Kantorowicz, Jadwiga Szymura, Justyna Kusmierczyk, Maria Lipowska, Zbigniew Szygula

**Affiliations:** 1 Department of Physiology and Biochemistry, University of Physical Culture, 31-571 Cracow, Poland; justyna.kusmierczyk@awf.krakow.pl; 2 Medical Institute, Academy of Applied Sciences, 34-400 Nowy Targ, Poland; malgorzata.kantorowicz@ans-nt.edu.pl; 3 Department of Clinical Rehabilitation, University of Physical Culture, 31-571 Cracow, Poland; jadwiga.szymura@awf.krakow.pl; 4 Laboratory of Biochemistry and Molecular Biology, University of Physical Culture, 31-571 Cracow, Poland; maria.lipowska@awf.krakow.pl; 5 Department of Sports Medicine and Human Nutrition, University of Physical Culture, 31-571 Cracow, Poland; zbigniew.szygula@awf.krakow.pl

**Keywords:** whole-body cryostimulation, metabolism, adipocytokines, meteorin-like hormone, irisin, asprosin, insulin resistance, menopause, exploratory study

## Abstract

Metabolic risk increases with age and may be accompanied by changes in adipocytokine secretion and insulin sensitivity. The primary exploratory aim of this prospective, uncon-trolled study was to determine whether repeated whole-body cryotherapy (WBC) changes circulating meteorin-like hormone (METRNL) concentrations in women of different ages. Secondary exploratory outcomes included irisin, asprosin, leptin, adiponectin, insulin, fasting blood glucose (FBG), lipid profile, homeostasis model assessment of insulin resistance (HOMA-IR), triglyceride–glucose index (TyG), atherogenic index of plasma (AIP), and the leptin/adiponectin ratio. A total of 48 women were divided into three groups: GR-60 (60–70 years, n = 14), GR-40 (40–50 years, n = 18), and GR-20 (20–30 years, n = 16). Participants underwent 30 WBC sessions, each lasting 3 min at minus 120 °C. Blood samples were collected before the first WBC session, after the 10th, 20th, and 30th sessions, and two weeks after the final session. Repeated WBC did not significantly change circulating METRNL or other adipocytokines in any age group. A preliminary decrease in insulin concentration and HOMA-IR was observed after 20 WBC sessions only in GR-60. These findings suggest that repeated WBC may be associated with favorable changes in selected insulin-resistance markers in older postmenopausal women; however, the absence of a non-WBC control group, small subgroup sizes, and the multiplicity burden preclude causal or therapeutic conclusions. Randomized controlled studies are needed to verify these exploratory observations.

## 1. Introduction

The development of metabolic complications increases with age and is associated, among other factors, with changes in adipocytokine and insulin secretion. In aging and metabolic disorders, increased concentrations of leptin and insulin and decreased concentrations of adiponectin and irisin have been reported [1,2,3,4,5,6]. The role of asprosin in healthy aging is less well established; current evidence is stronger in conditions characterized by insulin resistance, such as type 2 diabetes and polycystic ovary syndrome [2,4,6]. Changes in the leptin/adiponectin ratio are particularly important. Levels of the anti-inflammatory adiponectin may decrease with age, contributing to insulin resistance, whereas leptin levels may increase with greater white adipose tissue accumulation and visceral deposition, promoting leptin resistance and chronic low-grade inflammation [7].

Leptin regulates body weight by inhibiting food intake and stimulating energy expenditure through a feedback loop between adipocytes and the hypothalamus. However, in obesity, leptin levels are high because of the development of hypothalamic leptin resistance. In obesity, leptin has a pro-inflammatory effect, promotes preadipocyte differentiation, and stimulates the secretion of the pro-inflammatory cytokine TNF-α (tumor necrosis factor α) from adipocytes [8]. Adiponectin increases the sensitivity of myocytes and hepatocytes to insulin by inhibiting hepatic gluconeogenesis and increasing glucose uptake by skeletal muscles. It also increases the oxidation of free fatty acids, including in skeletal muscle. This hormone reduces blood concentrations of free fatty acids, triglycerides (TG), and glucose. Adiponectin also has anti-inflammatory properties [9,10]. Irisin may also have favorable effects on glucose homeostasis and insulin sensitivity by increasing energy expenditure, intensifying glycogenolysis and glycolysis, and reducing gluconeogenesis, adipogenesis, and lipid accumulation [11,12,13]. Asprosin is released by adipocytes and regulates glucose release from glycogen in hepatocytes [14]. It has also been reported to adversely affect muscle-cell sensitivity to insulin [15].

In women, sex hormones are important modulators of adipocytokine secretion at different ages [16]. Estradiol influences the production and secretion of leptin and adiponectin, depending partly on the predominance of estrogen receptor ERα over ERβ. In premenopausal women, higher estradiol concentrations positively correlate with leptin levels. At the same time, the sensitivity of hypothalamic cells to leptin increases. Adiponectin is found in higher concentrations in young women than in men [17,18,19,20]. The risk of metabolic diseases and their consequences increases with age, particularly after menopause, and may be related to disturbances in the ERα/ERβ ratio in adipose tissue. Hormonal fluctuations during perimenopause and persistent postmenopausal changes result in increased visceral fat content, leading to increased leptin concentrations independent of estrogen regulation [20]. Adiponectin concentrations may also increase and may correlate with loss of muscle mass and bone density, but its action may become unfavorable, which in older age is referred to as the “adiponectin paradox” [21]. The effect of progesterone on adipocytokines remains unclear. Some animal data indicate that progesterone inhibits adiponectin expression and activates leptin and resistin expression [22]. Studies in women aged 18–40 years confirm a positive correlation between leptin concentrations and estradiol and progesterone concentrations during the menstrual cycle [19].

Pro-inflammatory adipocytokines are involved in the pathogenesis of hypertension, lipid disorders, atherosclerosis, coronary heart disease, and type 2 diabetes [23]. Maintaining homeostasis and the proper integration of metabolic processes in response to internal and environmental stimuli that disturb carbohydrate–lipid balance requires the involvement of many mechanisms controlling metabolism, including those involving adipocytokines.

Reduced ambient temperature may modulate adipocytokine expression and secretion, but this response appears to depend on the adipose tissue depot, type of adipose tissue, temperature, and duration of cold exposure. Animal studies show that cold exposure can differently affect brown adipose tissue and distinct white adipose tissue depots, including epididymal and subcutaneous fat. For example, acute cold exposure in rats down-regulated leptin mRNA expression in both interscapular brown adipose tissue (iBAT) and epididymal white adipose tissue (eWAT), whereas adiponectin mRNA expression decreased only in iBAT and resistin mRNA expression remained unchanged [24]. Other animal studies have shown that cold exposure may reduce leptin expression across several adipose tissue depots, while adiponectin responses are more depot-specific and time-dependent, with transient or divergent changes in iBAT, eWAT, and subcutaneous white adipose tissue (sWAT) [25,26,27]. Importantly, changes in adipokine gene or protein expression within adipose tissue are not always reflected by parallel changes in circulating concentrations [24,25]. In humans, acute cold exposure has been associated with increased energy expenditure, BAT activity, glucose uptake, insulin sensitivity, and non-esterified fatty acid metabolism [28]. These findings indicate that adipose tissue responses to cold are complex and depot-specific, and that results from animal models of prolonged cold exposure should be translated cautiously to brief, intermittent WBC protocols in humans.

Collectively, these findings highlight the complexity of adipose tissue hormonal responses to cold exposure and indicate that modulation of adipocytokine expression and circulating concentrations may contribute to metabolic adaptations. Current evidence suggests that short-term but repeated whole-body exposure to cryogenic temperatures (whole-body cryotherapy, WBC) may influence metabolic and inflammatory markers in humans; however, the magnitude and clinical relevance of these effects require cautious interpretation, especially in uncontrolled studies [5,29,30,31,32,33,34].

Exposure to cryogenic temperatures (≤−110 °C) has been associated in previous studies with changes in inflammatory markers and hormones related to carbohydrate and lipid metabolism [29,30,31]. In menopausal women with elevated fasting blood glucose, metabolic syndrome, or type 2 diabetes, WBC has been reported to reduce fasting asprosin and glucose concentrations [5,34]. WBC-related induction of irisin secretion has also been described [31]. Because irisin is linked to white adipose tissue browning and correlates with meteorin-like hormone (METRNL), METRNL may be a relevant but still poorly characterized component of the endocrine response to cold exposure [35,36,37,38,39].

METRNL is a secretory protein with 46% homology to the neurotrophic Meteorin. Unlike Meteorin, which is brain-specific, METRNL is abundant in metabolism-related organs and barrier tissues, and its expression varies with physiological and pathological contexts [40]. Initially described as an adipokine that enhances insulin sensitivity, promotes adipose tissue browning, and increases energy expenditure, METRNL has also been linked to cardiometabolic and inflammatory processes [36].

METRNL has been shown to increase aerobic metabolism in skeletal muscle, influence the browning of white adipose tissue, stimulate the oxidation of free fatty acids, and regulate inflammation and insulin resistance [35,36,41]. Serum METRNL concentrations have been negatively correlated with metabolic parameters, including body mass index (BMI), total cholesterol (T-CHOL), and low-density lipoprotein cholesterol (LDL-C), as well as inflammatory markers, including high-sensitivity C-reactive protein (CRP) and proinflammatory interleukins. METRNL concentration has also been reported to be negatively related to the number of narrowed vessels and the severity of coronary artery disease [42].

In animal models, acute cold exposure has been shown to increase METRNL gene expression in adipose tissue, with the greatest kinetics occurring in BAT [35]. Longer exposure to cold also increased circulating METRNL concentrations in experimental animals [35]. These data suggest a potential role of METRNL in physiological adaptation to cold. However, evidence from animal models based on prolonged cold exposure cannot be directly translated to brief intermittent WBC in humans. To our knowledge, the effect of repeated WBC on circulating METRNL concentrations in humans has not yet been established.

The primary exploratory aim of our study was to determine whether 10, 20, and 30 WBC sessions change circulating METRNL concentration in healthy women of different ages. Secondary exploratory aims were to assess changes in irisin, asprosin, adiponectin, leptin, insulin, markers of carbohydrate–lipid metabolism, and calculated indices of insulin resistance and atherogenic risk, and to examine whether these responses differed between women aged 20–30, 40–50, and 60–70 years. Women were included because aging and menopause are associated with changes in estrogen status, body composition, visceral adiposity, waist circumference, waist–hip ratio (WHR), BMI, leptin concentration, glucose metabolism, lipid metabolism, and chronic low-grade inflammation [43]. Therefore, the study was designed to explore whether repeated WBC is associated with changes in adipocytokines and metabolic markers across different female age groups, without making a priori causal or therapeutic assumptions.

The physiological effects of WBC result from autonomically regulated cutaneous vasoconstriction during exposure to extreme cold, followed by dilation and reperfusion [44]. This is reflected in a rapid decrease in skin temperature during treatment and a two-phase return towards baseline after its completion. These changes depend on age, sex, and fat content. Faster heat loss, manifested by a rapid decrease in skin temperature, has been observed in older women with increased BMI [44]. Therefore, a different response may be expected in postmenopausal women, although such differences cannot be attributed to age alone. Participants had no medical contraindications to WBC [45] and were asked not to modify their lifestyle, including physical activity [46].

Despite evidence that cold exposure may modulate adipose tissue endocrine activity, it remains unclear whether brief, intermittent WBC alters circulating METRNL concentrations in humans. Existing evidence for cold-induced METRNL responses comes mainly from animal models of prolonged cold exposure, whereas data from repeated WBC protocols in women are lacking. Therefore, the primary exploratory aim of this study was to determine whether repeated WBC is associated with changes in circulating METRNL concentration in women of different ages. Secondary exploratory aims were to assess changes in selected adipocytokines and insulin-resistance markers.

We hypothesized that repeated exposure to cryogenic temperatures may be associated with changes in selected adipocytokines and insulin-resistance markers, with the largest response expected in older postmenopausal women. Given the prospective, uncontrolled, and exploratory design, this study was considered hypothesis-generating rather than confirmatory.

## 2. Results

### 2.1. Participants Characteristics

The age, BMI, and results of complete blood count and HbA1c of the participants are presented in Table 1.

As expected from the study design, the GR-20, GR-40, and GR-60 groups differed significantly in age (*p* < 0.05). Both the GR-60 and GR-40 groups had significantly higher HbA1c values than GR-20 (*p* < 0.05). In addition, GR-60 had significantly higher BMI and hematocrit values than GR-20 (*p* < 0.05). The monocyte count in GR-40 was significantly lower than in GR-20 (*p* < 0.05). These baseline differences indicate that the age groups were not metabolically identical at study entry.

### 2.2. Markers of Carbohydrate and Lipid Metabolism

The ANOVA analysis showed statistically significant effect of the main factor, Group, with a large effect size on FBG (*p* < 0.001, F = 12.05, η^2^ = 0.36), T-CHOL (*p* = 0.005, F = 6.03, η^2^ = 0.22), LDL-C (*p* = 0.017, F = 4.50, η^2^ = 0.18), TyG (*p* = 0.001, F = 9.08, η^2^ = 0.30), AIP (*p* = 0.014, F = 4.74, η^2^ = 0.18) and Lept/Adipo (*p* < 0.007, F = 5.54, η^2^ = 0.20). A trend of intergroup differences in TG (*p* = 0.063, F = 2.95) and leptin concentration (*p* = 0.061, F = 2.97) with a medium effect size (η^2^ = 0.12) was also demonstrated. There were no differences (*p* > 0.05) between groups in HDL-C, METRNL, irisin, asprosin, insulin and adiponectin concentration as well as HOMA-IR (Table 2 and Table 3).

The ANOVA analysis did not show a statistically significant effect of the main factor WBC on the concentration of the analyzed variables, except for the effect on FBG concentration, with a medium effect size (*p* = 0.032, F = 2.71, η^2^ = 0.06) (Table 2 and Table 3).

A significant effect of the Group x WBC interaction was observed for changes in irisin concentration (*p* = 0.001, F = 3.38, η^2^ = 0.14 large), insulin (*p* = 0.045, F = 2.04, η^2^ = 0.10 medium), and HOMA-IR (*p* = 0.039, F = 2.10, η^2^ = 0.10 medium) (Table 3). These interaction effects were obtained in unadjusted analyses. Because several biomarkers and comparisons were examined simultaneously, these findings should be interpreted as exploratory and not as confirmatory evidence of a WBC effect.

#### 2.2.1. Group Comparison

Before starting WBC, as a result of post hoc analysis, significantly higher concentrations of FBG (*p* = 0.001, ES = 1.49 large), T-CHOL (*p* = 0.002, ES = 1.24 large), LDL-C (*p* = 0.019, ES = 0.85 large), TG (*p* = 0.001, ES = 1.36 large) and leptin (*p* = 0.013, ES = 0.89 large), as well as significantly higher levels of TyG (*p* = 0.001, ES = 1.48 large) and AIP (*p* = 0.030, ES = 0.94 large), were found in the GR-60 group compared to the GR-20 group (Table 2 and Table 3). Before the WBC, insulin concentrations, HOMA-IR level and Lept/Adipo ratio in GR-60 were significantly higher compared to GR-20 (*p* = 0.017, ES = 0.35 small; *p* = 0.004, ES = 0.54 medium; *p* = 0.021, ES = 0.50 medium, respectively) and compared to GR-40 (*p* = 0.041, ES = 0.67 medium; *p* = 0.036, ES = 0.73 medium; *p* = 0.005, ES = 0.62 medium, respectively) (Table 3).

GR-40 was characterized by significantly higher concentrations of FBG (*p* = 0.021, ES = 0.65 medium), T-CHOL (*p* = 0.001, ES = 1.37 large), and LDL-C (*p* = 0.007, ES = 1.18 large), and a significantly higher level of TyG (*p* = 0.021, ES = 0.88 large) compared to GR-20 (Table 2).

Throughout the entire period of WBC, as well as two weeks after the end of WBC (post hoc analysis), FBG concentration and TyG and AIP values were significantly higher in GR-60 than in GR-20 (*p* < 0.05, ES > 0.8 large). The *p*-values and effect sizes (ESs) of these differences after 10 WBC, 20 WBC, and 30 WBC sessions and two weeks after the end of WBC, respectively (Table 2), were as follows: for FBG: *p* = 0.001, ES = 0.87; *p* = 0.001, ES = 1.06; *p* < 0.001, ES = 1.39; and *p* = 0.001, ES = 1.42; for TyG: *p* = 0.001, ES = 1.58; *p* = 0.001, ES = 1.60; *p* < 0.001, ES = 2.01; and *p* = 0.001, ES = 1.49; and for AIP: *p* = 0.006, ES = 1.18; *p* = 0.003, ES = 1.39; *p* = 0.001, ES = 1.58; and *p* = 0.022, ES = 0.98.

FBG concentration and TyG values in GR-60 were also significantly higher (*p* < 0.05) than in GR-40, but the effect sizes were small or medium, respectively. The *p*-values and effect sizes for FBG differences were: *p* = 0.044, ES = 0.26, small after 10 WBC sessions; *p* = 0.036, ES = 0.28, small after 20 WBC sessions; *p* = 0.049, ES = 0.56, medium after 30 WBC sessions; and *p* = 0.001, ES = 0.43, small two weeks after the end of WBC. For TyG they were: *p* = 0.044, ES = 0.76, medium; *p* = 0.036, ES = 0.74, medium; and *p* = 0.049, ES = 0.71, medium, respectively (Table 2).

Additionally, GR-60 compared to GR-20 showed significantly higher concentrations of LDL-C after 10 WBC sessions (*p* = 0.017, ES = 0.81), T-CHOL after 10 WBC sessions (*p* = 0.007, ES = 0.97) and after 30 WBC sessions (*p* = 0.022, ES = 0.96), and TG concentration after 10 WBC sessions (*p* = 0.005, ES = 1.36), after 20 WBC sessions (*p* = 0.008, ES = 1.59), and after 30 WBC sessions (*p* = 0.007, ES = 1.68), with a large effect size (Table 2).

During the entire WBC therapy period (post hoc analysis), after 10 WBC (*p* = 0.001, ES = 1.44), 20 WBC (*p* = 0.045, ES = 0.74) and 30 WBC sessions (*p* = 0.001, ES = 1.29), as well as 2 weeks after its completion (*p* = 0.011, ES = 0.96), the GR-40 group was characterized by significantly higher T-CHOL concentration, significantly higher LDL-C concentration after 10 WBC sessions (*p* = 0.001, ES = 1.37), after 30 WBC sessions (*p* = 0.003, ES = 1.10) and 2 weeks after the completion of WBC therapy (*p* = 0.023, ES = 0.94), and significantly higher TyG value after 30 WBC sessions (*p* = 0.006, ES = 1.07) and 2 weeks after the completion of WBC therapy (*p* = 0.008, ES = 1.01), compared to GR-20. The effect size of these intergroup differences was large (Table 2).

Additionally, leptin concentrations in GR-60 were significantly higher than in GR-20 after 30 WBC sessions (*p* = 0.018, ES = 0.84 large) and two weeks after completing therapy (*p* = 0.035, ES = 0.70 medium). Two weeks after completing therapy, the GR-60 also had higher HOMA-IR values than GR-20 (*p* = 0.033, ES = 1.01 large), as well as a higher Lept/Adipo compared to both GR-20 (*p* = 0.030, ES = 0.61 medium) and GR-40 (*p* = 0.014, ES = 0.78 medium) (Table 3).

#### 2.2.2. Effects of Whole-Body Cryotherapy

After 20 WBC treatments, the reduction in insulin concentration (*p* = 0.03) and HOMA-IR (*p* = 0.04) in GR-60 was statistically significant, and the effect sizes were medium (ES = 0.74) and large (ES = 0.80), respectively (Supplementary Materials Table S2). Because these findings were observed in one small subgroup, were not supported by a parallel non-WBC control arm, and were evaluated in the context of multiple outcomes, they should be interpreted as preliminary exploratory signals rather than evidence of a causal WBC effect.

For the remaining variables in the GR-60, GR-40, and GR-20 groups, regardless of the number of treatments, no statistically significant within-group changes in concentration (*p* > 0.05) were observed after WBC (Supplementary Materials Tables S1 and S2).

### 2.3. Correlations

Correlations were determined between the concentration of insulin and selected adipocytokines such as METRNL, irisin, asprosin, adiponectin and leptin in the blood and the level of indicators of carbohydrate–lipid metabolism (FBG, HbA1c, HOMA-IR, T-CHOL, LDL-C, HDL-C, TG, AIP and TyG), as well as BMI, in healthy women for the entire group and by age. The complete set of correlations, together with their power values, is provided in Data S1 (Supplementary Materials). These analyses were considered exploratory and were not used to infer causality.

It was found that regardless of age (total cohort, n = 48), there was a statistically significant, positive correlation between insulin concentration and the HOMA-IR (r = 0.98, high; *p* < 0.001; 1-β = 1.00) and HbA1c (r = 0.56, moderate; *p* < 0.001; 1-β = 0.99), between leptin concentration and BMI (r = 0.54, moderate; *p* < 0.001; 1-β = 0.98), and between adiponectin, irisin and asprosin concentration and HDL-C (r = 0.60, moderately high; *p* < 0.001; 1-β = 1.00 and r = 0.30, low; *p* < 0.040; 1-β = 0.54 and r = 0.32, low; *p* < 0.025; 1-β = 0.61; respectively).

A negative correlation, regardless of age (total cohort, n = 48), was found between the concentration of irisin and HbA1c (r = −0.37, low; *p* < 0.010; 1-β = 0.73), HOMA-IR (r = −0.36, low; *p* < 0.011; 1-β = 0.72) and AIP (r = −0.33, low; *p* < 0.022; 1-β = 0.63), as well as between the concentration of adiponectin and TG (r = −0.30, low; *p* < 0.040; 1-β = 0.54) and the AIP level (r = −0.45, moderate; *p* = 0.010; 1-β = 0.90).

When the participants were divided into groups according to age, only in the GR-60, statistically significant correlations (*p* < 0.05) were found between the concentration of METRNL and indicators of carbohydrate–lipid metabolism. In the GR-60 group, negative correlations were found between METRNL concentration and FBG (r = −0.77, moderately high; *p* = 0.001; 1-β = 0.92), TG (r = −0.59, moderate; *p* = 0.026; 1-β = 0.62), AIP (r = −0.63, moderately high; *p* = 0.016; 1-β = 0.69), and TyG (r = −0.72, moderately high; *p* = 0.003; 1-β = 0.86). In the GR-60, there were also significant, negative moderately high correlations between the concentration of irisin and FBG (r = −0.66, *p* = 0.010; 1-β = 0.75) and HOMA-IR (r = −0.63, *p* = 0.016; 1-β = 0.69), significant positive correlations between insulin concentration and HbA1c (r = 0.58, moderate; *p* = 0.030; 1-β = 0.59) and HOMA-IR (r = 0.98, high; *p* < 0.001; 1-β = 1.00), and moderately high correlations between asprosin and adiponectin concentration and HDL-C (r = 0.75 and r = 0.65; *p* = 0.002 and *p* = 0.011; 1-β = 0.89 and 1-β = 0.74; respectively).

In the GR-40, moderately high correlations were found between insulin level and HbA1c (r = 0.61, *p* = 0.007; 1-β = 0.79), TG (r = 0.79, *p* < 0.001; 1-β = 0.99), AIP (r = 0.78, *p* < 0.001; 1-β = 0.98) and TyG (r = 0.75, *p* < 0.001; 1-β = 0.96), as well as high correlations with HOMA-IR (r = 0.99, *p* < 0.001; 1-β = 1.00). In the GR-40, a positive moderately high correlation was also found between BMI and insulin concentration (r = 0.78, *p* < 0.001; 1-β = 0.98) and leptin concentration (r = 0.63, *p* = 0.005; 1-β = 0.82), as well as a negative moderately high correlation between asprosin concentration and HbA1c (r = −0.70, *p* = 0.001; 1-β = 0.92). A moderate positive correlation was also observed between leptin concentration and HOMA-IR (r = 0.49, *p* = 0.039; 1-β = 0.55) and a moderate negative correlation between adiponectin concentration and BMI (r = −0.52, *p* = 0.025; 1-β = 0.62) in the GR-40 study.

In the GR-20, a positive correlation was found between insulin concentration and HbA1c (r = 0.60, moderately high; *p* = 0.013; 1-β = 0.71) and HOMA-IR (r = 1.00, high; *p* < 0.001; 1-β = 1.00). A positive moderately high correlation was found between irisin concentration and HDL-C (r = 0.66, *p* = 0.005; 1-β = 0.82), alongside, at the same time, a negative moderately high correlation with AIP (r = −0.60, *p* = 0.015; 1-β = 0.70) and negative moderate correlation with HbA1c (r = −0.57, *p* = 0.021; 1-β = 0.65). Adiponectin concentration correlated positively with HDL-C (r = 0.73, moderately high; *p* = 0.001; 1-β = 0.91) and FBG (r = 0.60, moderately high; *p* = 0.015; 1-β = 0.71), but negatively with AIP (r = −0.53, moderate; *p* = 0.036; 1-β = 0.56). In GR-20, a negative correlation was found between leptin concentration and HbA1c (r = −0.55, moderate; *p* = 0.027; 1-β = 0.61).

## 3. Discussion

The main finding of this prospective, uncontrolled study was that repeated WBC did not significantly change circulating METRNL or other analyzed adipocytokines in any age group. The only favorable metabolic signal was a decrease in insulin concentration and HOMA-IR after 20 WBC sessions in the oldest group of women. This decrease was numerically visible after 10 WBC sessions and reached statistical significance after 20 sessions. The positive effect for the insulin and HOMA-IR signal after 20 WBC sessions, without persistence after 30 sessions, suggests that the response may not be linear or cumulative. This pattern may reflect a transient or adaptive response to repeated cold exposure. However, because this finding was observed in a small subgroup, without a non-WBC control group and in the context of multiple tested outcomes, it should be interpreted as preliminary and hypothesis-generating.

Although the study included women without diagnosed diabetes, eight participants, including five in GR-60 (36%) and three in GR-40 (17%), had baseline FBG values suggestive of impaired fasting glucose (>5.5 mmol/L). After 30 WBC sessions, seven of these women had FBG values below this threshold, including all three women in GR-40 and four women in GR-60. This observation is clinically interesting, but it should be interpreted cautiously because of the absence of a control group and the small number of participants with elevated baseline FBG.

In younger and middle-aged women, WBC did not induce statistically significant changes in adipocytokines or insulin-resistance markers. In GR-40, the positive correlations between insulin concentration and HbA1c, HOMA-IR, TG, TyG, AIP, and BMI may indicate an unfavorable metabolic profile in some participants. Although some changes were observed in a favorable direction, the present study does not allow conclusions regarding preventive effects of WBC in these age groups. The absence of clear responses may reflect lower baseline metabolic risk, menstrual-cycle-related variability, or insufficient sensitivity of next-morning fasting measurements to capture acute hormonal changes.

The decrease in insulin and HOMA-IR after 20 WBC sessions in GR-60 may indicate that older postmenopausal women with a less favorable baseline metabolic profile could be more responsive to repeated cold exposure. However, in view of the multiplicity burden, this finding should be treated as an exploratory signal only. Given the number of simultaneously analyzed biomarkers and indices, the possibility that the observed insulin and HOMA-IR findings represent false-positive results cannot be excluded. The *p*-values for these analyses were close to the conventional significance threshold and therefore should not be interpreted as evidence supporting an effect of WBC without replication in an adequately powered, controlled study with a pre-specified primary outcome. The present design does not allow us to determine with certainty whether this change was caused by WBC, time-dependent variation, or other unmeasured factors.

Asprosin and METRNL are relatively recently described peptide hormones involved in metabolic regulation [14,35]. Previous studies have shown that, in menopausal women, circulating asprosin may correlate positively with risk factors for metabolic disorders, including fasting glucose, atherogenic indices, and the leptin/adiponectin ratio [5]. In the present study, the positive association between asprosin and HDL-C in GR-60 and the negative association between asprosin and HbA1c in GR-40 should be interpreted cautiously because these were exploratory correlations in small subgroups. In the oldest group, higher METRNL concentrations were associated with lower FBG, TG, TyG, and AIP values. However, these associations do not imply causality and require confirmation in larger cohorts.

Previous studies have reported changes in selected peptide hormones related to carbohydrate and lipid metabolism after exposure to cryogenic temperatures [29,30,31]. For example, 10 WBC sessions increased circulating irisin concentration in inactive men with obesity, while no such effect was observed in active men with obesity [30]. In the case of adiponectin and leptin, the number of WBC sessions (1, 5, or 10) had no significant effect on circulating concentrations, regardless of physical activity levels in men with obesity [33]. However, leptin concentration was reported to decrease between the 10th and 20th WBC session in men with class I and II obesity, while it remained unchanged in men without obesity [47]. In the present study, we did not find any significant effect of WBC on adiponectin or leptin concentrations in women. We also found no significant increase in irisin concentration in any age group, regardless of the number of WBC sessions, in contrast to the increase previously observed in older women after 10 WBC sessions [31]. Similarly, although previous studies reported decreases in fasting asprosin and glucose concentrations after 20 WBC sessions in menopausal women with elevated fasting glucose [5], and in postmenopausal women with type 2 diabetes [34], we did not observe significant changes in asprosin concentration in the present cohort.

Differences between age groups in the observed responses after WBC may be partly related to hormonal status, fat-tissue distribution, baseline metabolic risk, and adiposity rather than age alone.

A positive correlation between METRNL and irisin and a negative correlation with visceral fat have previously been demonstrated in patients with type 2 diabetes. METRNL has been suggested to participate in adipose tissue metabolism by activating aerobic processes in myocytes, stimulating thermogenic pathways in brown adipose tissue, or inducing the secretion of myokines that may increase energy expenditure through increased utilization of glucose and free fatty acids [35,36,39,41]. However, in the present study, WBC did not significantly affect circulating METRNL concentrations in any age group. Therefore, our findings do not support the assumption that brief, repeated, whole-body exposure to cryogenic temperature induces a measurable increase in circulating METRNL in healthy women.

As previously demonstrated in animal models [35], cold-induced metabolic effects of METRNL may involve activation of eosinophils and secretion of IL-4 and IL-13, followed by macrophage polarization toward the M2 phenotype, adipocyte browning, and thermogenesis. However, the translational relevance of these mechanisms to humans undergoing WBC remains uncertain. Most evidence for cold-related METRNL induction comes from animal models exposed to prolonged cold, whereas WBC in humans is brief and intermittent, and acts through cryogenic temperatures. Moreover, tissue mRNA expression and circulating protein concentrations may be regulated differently. It is therefore possible that WBC does not meaningfully affect peripheral METRNL concentrations under the conditions used in the present protocol. It is also possible that any changes in circulating METRNL after WBC are short-lived and were not captured by the next-morning fasting blood sampling schedule.

To date, data on changes in circulating METRNL after WBC in humans remain limited. In the present study, the absence of a significant METRNL response may reflect insufficient stimulus duration, high interindividual variability, or limited statistical power within age subgroups. The lack of peripheral METRNL changes after WBC does not exclude local autocrine or paracrine effects in adipose tissue or skeletal muscle; however, such mechanisms remain hypothetical because gene expression, BAT activity, and WAT browning were not assessed.

The exploratory insulin and HOMA-IR signal observed in GR-60 may be discussed in the context of physiological responses to cold, which can vary according to age, hormonal status, and body composition. Previous work has shown that, under the same WBC procedure, women, individuals with higher BMI, and older adults may reach the target cold-induced skin temperature faster and maintain lower skin temperature during the rewarming phase [44]. This may be related to the thermal insulating properties of adipose tissue, which was reflected in the higher BMI observed in GR-60 compared with GR-40 and GR-20. Faster skin cooling may lead to earlier activation of skin thermoreceptors and may potentially influence energy expenditure and thermogenic pathways [48]. Additionally, a decline in estrogen concentration after menopause may reduce the thermogenic potential of brown adipose tissue and modify the adipokine secretion profile [20,49]. These mechanisms may partly explain different metabolic responses in older and younger women. However, because BAT activity, WAT browning, and gene expression were not assessed, these mechanisms remain hypothetical in the present study.

The dependence of cold-induced vasoconstriction and thermoregulatory responses on adipose tissue content suggests that individualized WBC procedures, including duration and temperature, may be needed to achieve comparable physiological responses across sex, age, and body-composition groups [50,51]. In the present study, all participants underwent the same WBC procedure, regardless of age and BMI. Although GR-20 and GR-40 did not differ significantly in BMI, individual differences in body composition could have influenced the results. In addition, BMI was significantly higher in GR-60 than in the younger groups. Interindividual variability in physiological responses to WBC may partly explain the absence of statistically significant changes in most analyzed biomarkers. Therefore, our results should be interpreted with caution to avoid overestimating the metabolic effects of WBC, while still recognizing possible biological implications that require controlled confirmation.

Overall, this study provides preliminary information on endocrine and metabolic responses to repeated WBC in women of different ages, but several limitations must be emphasized.

### Study Limitations

The principal limitation of this study is the absence of a parallel non-WBC control group. Consequently, causal inference is not supported, and the observed changes cannot be attributed specifically to WBC. Repeated-measurement effects, time-dependent variation, behavioral changes during study participation, and hormonal variability may have influenced the results. The study also included only Caucasian women and relatively small age subgroups, which limits generalizability and statistical power, particularly for subgroup and correlation analyses. In addition, the groups differed at baseline in age-related metabolic characteristics, including BMI, HbA1c, fasting glucose, lipid markers, insulin, and HOMA-IR; therefore, the apparent age-related response may also reflect baseline metabolic risk, menopausal status, or adiposity rather than age alone. The study was not powered to confirm small biomarker effects after correction for multiple testing. Therefore, the insulin and HOMA-IR results should be considered exploratory.

Another important limitation is the timing of blood collection. Samples were collected the next morning, approximately 14–18 h after afternoon WBC sessions; the protocol assessed fasting next-day responses rather than acute post-WBC hormonal peaks. Short-lived changes in hormones such as irisin, asprosin, or METRNL may have been missed. Previous studies have shown that a single exposure to cryogenic temperatures can induce rapid intracellular and peripheral molecular changes during the rewarming phase after WBC [44], including modulation of inflammation, adipokine levels, and oxidative stress [30,52,53,54,55,56,57,58]. An increase in irisin concentration has been observed one hour after a single WBC session [52]. Many metabolic and endocrine responses to low temperature may therefore be rapid, transient, or delayed. Our protocol, with assessments after the 10th, 20th, and 30th WBC session and after two weeks of follow-up, allowed evaluation of cumulative and delayed responses, but may not have captured immediate transient effects. Future studies should include both acute and repeated-sampling protocols, particularly when evaluating less well-characterized cytokines such as METRNL.

Dietary diaries and IPAQ monitoring were used to describe lifestyle stability during participation; however, they do not eliminate confounding in an uncontrolled design. Future studies should include randomized sham, waitlist, or non-WBC control groups to separate treatment-related effects from time-dependent changes.

Due to the study duration and differences in menstrual-cycle length, serial blood sampling in GR-20 and GR-40 could not be synchronized to the same menstrual-cycle phase. To minimize the influence of menstrual-cycle hormones on the outcomes [16,19,59], participants began WBC in either the follicular or luteal phase, but subsequent blood collections were not phase-controlled, and estradiol and progesterone were not measured. This may have introduced systematic variability in leptin, adiponectin, and other adipocytokines and may have contributed to null findings in the younger groups. Future protocols should include repeated measurement of estradiol and progesterone.

In summary, future studies should compare metabolic responses to cryogenic temperatures in women and men of different ages. In addition to circulating adipocytokines, future protocols should assess adipocyte and/or myocyte gene expression, body composition, waist circumference, visceral adiposity markers, BAT activity, and WAT browning, and should include appropriate non-WBC control groups.

## 4. Materials and Methods

The study involved 48 Caucasian women in three age groups, i.e., GR-20: 20–30 years (n = 16), GR-40: 40–50 years (n = 18) and GR-60: 60–70 years old (n = 14), who were subjected to 30 WBC sessions (3 min each, at −120 °C), performed at the Małopolska Cryotherapy Center in Krakow in six series of 5 treatments with a break on the weekend.

Once the age requirement was met, exclusion conditions included medical contraindications to WBC [45], participation in WBC within the last 6 months or medical conditions requiring continuous pharmacotherapy.

During project implementation, the women did not change their diet or physical activity, which was monitored using food diaries and the International Physical Activity Questionnaire (IPAQ)—short version [46].

Inclusion in the study took place after medical qualification, which included medical interview, physical examination and analysis of blood count, lipid profile, glucose level and glycated hemoglobin (HbA1c). The interview also included a gynecological interview regarding the menstrual cycle (menarche age, cycle length, regularity of the menstrual cycle, beginning of the last menstrual bleeding), number of pregnancies and childbirths, gynecological and obstetrical surgeries, and use of contraceptives as well as hormone replacement therapy. Women classified to groups GR-20 and GR-40 were characterized by a regular menstrual cycle of 27–32 days and did not use contraceptives. None of the women in group GR-20 had given birth to date. Women in group GR-40 had given birth at least 10 years before starting the study. Women in group GR-60 were in menopause (at least 12 months without a menstrual cycle) but did not use hormone replacement therapy.

Women in the GR-20 and GR-40 groups started the WBC procedures in the follicular phase (FP), 5–10 days after the onset of menstrual bleeding, or in the luteal phase (LP), 20–25 days after the onset of menstrual bleeding of the current or the next menstrual cycle. In the FP, WBC procedures were started by 8 women in the GR-20 and 8 women in the GR-40, whereas in the luteal phase, WBC procedures were started by 8 women in the GR-20 and 10 women in the GR-40, who completed the entire study cycle.

During the study, fasting glucose, lipid profile, and selected hormone concentrations were assessed at baseline and after the 10th, 20th, and 30th WBC session. Biochemical determinations were performed in the Laboratory of Biochemistry and Molecular Biology at the University of Physical Culture in Krakow.

### 4.1. Whole-Body Cryotherapy Procedure

Each of the volunteers participated in 30 WBC procedures. The procedures were performed on weekdays in the afternoon (3:00 p.m.–5:00 p.m.), in six series of 5 WBC procedures performed daily from Monday to Friday, excluding weekends (Saturday, Sunday). WBC treatments were carried out under the supervision of a doctor and a physiotherapist in a stationary cryogenic chamber Bamet KN-1 (Bamet, Wielka Wieś, Poland). During WBC, subjects spent 30 s in the atrium (−60 °C) and then 3 min in the main chamber (−120 °C). During treatment, the participants (maximum 4 people at a time) walked calmly “in circles”, one by one, and the direction of walking was changed every 30 s on a signal. The women were dressed in shorts, a sleeveless T-shirt, socks covering the ankle and knee joints, gloves, a hat or a headband covering the ears, and clogs with a wooden sole. The mouth and nose were covered with a surgical mask with an additional layer of gauze. Women took off jewelry, glasses and contact lenses. They were without makeup and had dry skin without any cosmetic products.

Communication with participants during the treatments was ensured via an integrated audio system and observation through thermal windows located in the main chamber door. The chamber temperature was continuously monitored, while the air was dried to maintain stable conditions. Oxygen concentration was kept constant at 21–22% and continuously monitored using two independent oxygen sensors (EurOx.O_2_ G/E, Cracow, Poland).

### 4.2. Biochemical Indices

#### 4.2.1. Blood Collection and Biochemical Analysis

**Figure 1:**
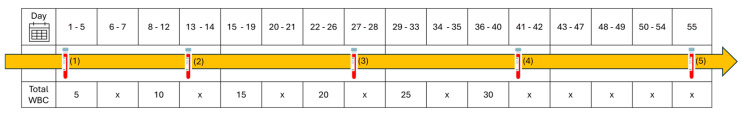
Fasting blood collection schedule, indicating subsequent days of study participation and the number of whole-body cryotherapy (WBC) treatments.

Venous blood was collected 5 times, always on an empty stomach, between 7:00 and 8:00 a.m. (Figure 1):

(1) Before 1 WBC—on the day of the first WBC procedure (day 1);
(2) After 10 WBC—in the morning, the next day after 10 WBC procedures (day 13);
(3) After 20 WBC—in the morning, the next day after 20 WBC procedures (day 27);
(4) After 30 WBC—in the morning, the next day after 30 WBC procedures (day 41);
(5) After 2 weeks—in the morning, two weeks after the 30 WBC procedure (day 55).

Venous blood was collected from the antecubital fossa region in the sitting position on the blood collection chair, after a 5 min rest in this place, using the BD Vacutainer^®^ vacuum system (Becton Dickinson, Franklin Lakes, NJ, USA), observing aseptic and antiseptic principles. During the rest period, information regarding the research was provided.

Participants were asked to fast for 12 h prior to blood collection and to sleep for at least 8 h. For 24 h prior to blood collection, participants refrained from physical exertion, except for walking. Blood collection took place 1–2 h after waking up. All venous blood samples were collected into anonymized tubes labeled with an individual code consisting of numbers and letters. Codes were assigned in the order of enrollment, without any specific indication of the participant’s age group. After insertion of the vacuum system needle and blood appearing in the transparent part of the cannula, the tourniquet was removed, allowing blood to flow into tubes, which were filled to the level indicated by a special marker. The same order of blood collection tubes was maintained each time: one with a clot activator, one with K2EDTA and a protease inhibitor, and then one with K2EDTA and a glycolysis inhibitor. After collection, the blood was gently mixed by inverting the tube several times.

Blood samples collected for fasting blood glucose (FBG) determinations were drawn into tubes containing K2EDTA with glycolysis inhibitors (sodium fluoride and potassium oxalate). For hormone analyses (excluding insulin), blood was collected into K2EDTA tubes with the protease inhibitor aprotinin (0.6 TIU/mL of blood). Immediately after collection, all samples were centrifuged at RCF 1000× *g* for 15 min at 4 °C (MPW-351R, MPW Med. Instruments, Warsaw, Poland).

For the determination of insulin, T-CHOL, high-density lipoprotein cholesterol (HDL-C), and TG, blood was collected into clot activator tubes. These were kept at room temperature (20–22 °C) for 20 min to allow clotting, and then centrifuged under the same conditions as described above.

Prior to analysis, plasma and serum samples were stored at −70 °C (ULF 390 Arctiko low-temperature freezer, Esbjerg, Denmark).

FBG and TG concentrations were determined using an enzymatic method, in accordance with the manufacturer’s manual, using reagents dedicated to the Cobas c 701/702 chemistry analyzer (Roche Diagnostics GmbH, Mannheim, Germany). The measuring range of the test for glucose (GLUC3) was 2–750 mg/dL, for T-CHOL (CHOL2) was 3.86–800 mg/dL, for HDL-C (HDLC3) was 3–120 mg/dL, and for TG (TRIGL), 8.85–885 mg/dL.

Insulin and other hormones were quantified using enzyme-linked immunosorbent assays (ELISAs), with absorbance readings performed on a Spark^®^ multimode microplate reader (Tecan, Grödig, Austria). The following assays were used according to the manufacturers’ protocols:

− Insulin: DCM076-8 kit (DiaMetra, Segrate, Italy), detection range 2.1–200 μIU/mL; intra-assay CV < 3.6%, inter-assay CV < 6.5%.
− Asprosin: Nori^®^ Human Asprosin ELISA Kit GR111426 (Genorise, Glen Mills, PA, USA), detection range 1.5–100 ng/mL; intra-assay CV < 6%, inter-assay CV < 9%.
− Irisin: Human Irisin ELISA Kit RAG018R (BioVendor, Karasek, Czech Republic), detection range 0.001–5 μg/mL; intra-assay CV < 8.2%, inter-assay CV < 9.7%.
− Leptin: Human Leptin ELISA Kit RD191001100 (BioVendor), detection range 0–50 ng/mL; intra-assay CV < 7.6%, inter-assay CV < 6.7%.
− Adiponectin: Human Adiponectin ELISA Kit RD191023100 (BioVendor), detection range 2–150 ng/mL; intra-assay CV < 4.4%, inter-assay CV < 6.6%.
− Meteorin-like hormone: Human Meteorin-like/METRNL ELISA Kit DY7868-05 (R&D Systems, Minneapolis, MN, USA), detection range 15.6–1000 pg/mL; intra-assay CV < 8.0%, inter-assay CV < 5.7%. Samples were analyzed according to the manufacturer’s protocol and concentrations were expressed in ng/mL after unit conversion. Samples with concentrations exceeding the assay’s upper detection limit were diluted and re-assayed. No METRNL values were below the assay detection limit.

Results were calculated from the standard curve, with verification based on the concentration of a standard control sample.

The laboratory analyst received only coded samples and was not informed about the study protocol details. Biochemical measurements were thus performed under blinded conditions with respect to participant identity, group allocation, and sampling time point. The same biochemical marker was always determined by the same person in a certified analytical laboratory. Samples from the same participant were analyzed in the same series, using a single set of reagents, and maintaining the same ambient temperature conditions. The same laboratory equipment was used.

#### 4.2.2. Calculated Indices

The following indices were calculated—HOMA-IR (homeostasis model assessment of insulin resistance), TyG (triglyceride–glucose index), AIP (atherogenic index of plasma), and LDL-C—according to the formulas below:

HOMA-IR = insulin (mU/mL) × FBG (mmol/L)/22.5(1)

TyG = lnTG × FBG/2(2)

AIP = log_10_(TG/HDL-C) (3)

LDL-C = T-CHOL − (HDL-C + TG/2.2)(4)

### 4.3. Statistical Analysis

**Figure 2:**
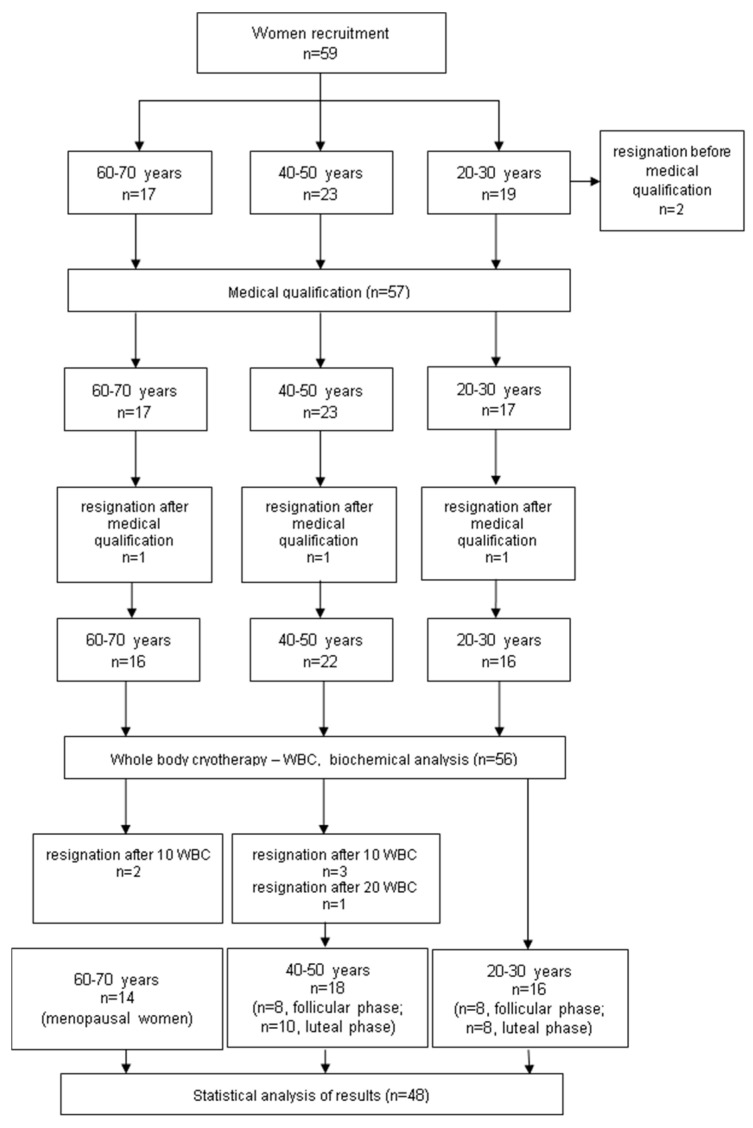
Flow chart of the procedure of participants’ qualifications.

The minimum required sample size for repeated measures analysis of variance (3 groups, 5 measurements) was calculated using G*Power 3.1.9.6 (Franz Faul, University of Kiel, Kiel, Germany). An expected medium effect size of 0.25 (according to Cohen’s convention) was assumed for the compared variables. With statistical power set at 0.95 and α = 0.05, the total sample size was estimated at 39 participants. In the present study, complete datasets from 48 women were included in the analysis (Figure 2).

All statistical analyses were performed using STATISTICA 13.3 software (StatSoft, Inc., Tulsa, OK, USA). Statistical significance was accepted at *p* < 0.05. The normality of data distribution was tested with the Shapiro–Wilk test, and the homogeneity of variances was assessed with Levene’s test. The primary exploratory outcome was the change in circulating METRNL concentration. Secondary exploratory outcomes included changes in irisin, asprosin, leptin, adiponectin, insulin, FBG, lipid profile, HOMA-IR, TyG, AIP, and the leptin/adiponectin ratio. Because no universally accepted reference ranges or clinically validated cut-off values are available for circulating METRNL, METRNL was interpreted as a relative change over time rather than as normalization or clinically defined improvement.

For single-time-point comparisons between groups, one-way analysis of variance (ANOVA) was applied to normally distributed variables, whereas the Kruskal–Wallis test was used for non-normally distributed variables, followed, when appropriate, by Student’s t-test or the Mann–Whitney U test, respectively.

To evaluate the effects of WBC on changes in the analyzed variables across age groups, a two-way repeated measures ANOVA was conducted, assessing the main effects of Group (GR-20, GR-40, GR-60), WBC (WBC count), and the Group × WBC interaction. When significant effects were found, Bonferroni post hoc comparisons were performed to control for type I error in multiple comparisons. Effect sizes (ESs) for the repeated measures ANOVA were reported as partial eta squared (η^2^) and interpreted as small (0.010–0.059), medium (0.060–0.139), or large (≥0.14). Additionally, the statistical power (1-β) was calculated for significant tests to assess the probability of detecting true effects given the sample size. Given the number of biomarkers, time points, and group comparisons, the overall multiplicity burden was high; therefore, significant unadjusted findings were interpreted cautiously as exploratory unless supported by consistent patterns across analyses. No post hoc division of outcomes into smaller sets was used to reduce the multiplicity burden.

Post hoc comparisons between study time points were performed using Bonferroni correction. However, because several biomarkers and calculated indices were analyzed simultaneously, the overall multiplicity burden was acknowledged. Therefore, findings from secondary and exploratory outcomes were interpreted cautiously, with emphasis on effect sizes, biological plausibility, and the need for confirmation in controlled studies.

For changes after WBC, means and 95% confidence intervals (CIs) were calculated. Effect sizes between baseline and post-WBC values were determined using Cohen’s d, while inter-group differences were expressed as Hedges’ g (to account for unequal group sizes). Effect sizes were interpreted as none (<0.20), small (0.20–0.499), medium (0.50–0.799), or large (≥0.80).

Correlations between variables were assessed using Pearson’s or Spearman’s tests, depending on the data distribution. Correlations were considered statistically significant at *p* < 0.05. The statistical power of correlation analyses (1-β) was calculated post hoc using G*Power 3.1.9.6 (Franz Faul, University of Kiel, Germany), based on the observed correlation coefficients (r), the sample size (n), and the significance level (α = 0.05). A power < 0.80 was interpreted as indicating an increased risk of type II error (failure to detect an existing effect), whereas a power ≥ 0.80 suggested that the test had sufficient sensitivity to reliably confirm the observed correlation.

Correlation coefficients (r) were interpreted as follows: no correlation (r ≤ 0.19), low correlation (0.20 ≤ r ≤ 0.39), moderate correlation (0.40 ≤ r ≤ 0.59), moderately high correlation (0.60 ≤ r ≤ 0.79), and high correlation (r ≥ 0.80).

## 5. Conclusions

In this prospective, uncontrolled study, repeated WBC did not significantly change circulating METRNL or other analyzed adipocytokines in healthy women of different ages. Reduction in insulin concentration and HOMA-IR was observed after 20 WBC sessions only in the oldest group of postmenopausal women; however, this result should be interpreted as exploratory because of the lack of a non-WBC control group, small subgroup size, and multiplicity burden.

These findings should be interpreted as preliminary and hypothesis-generating. They do not establish a causal or therapeutic effect of WBC. Nevertheless, this exploratory metabolic signal suggest that older women with a less favorable baseline metabolic profile may represent an important target group for future randomized controlled studies with appropriate non-WBC control groups. From a translational perspective, future research should test whether repeated WBC may influence a broader immune-metabolic pathway involving improved adipokine profile, including increased adiponectin and reduced pro-inflammatory adipokines, improved insulin sensitivity, reduced systemic low-grade inflammation, decreased spleen-related immune activation, potentially reflected by reduced spleen activity or size, and reduced inflammatory signaling to the liver.

## Figures and Tables

**Table 1:** Age, body mass index, and baseline hematological and metabolic characteristics of participants.

Variable	GR-20 (a)	GR-40 (b)	GR-60 (c)	*p*-Value
Age (years)	20.81 ± 2.83	45.83 ± 3.24 ^a^	65.07 ± 3.22 ^a,b^	<0.01
BMI (kg/m^2^)	23.01 ± 3.98	25.24 ± 2.99	27.52 ± 2.56 ^a^	<0.01
Erythrocytes (10^6^/µL)	4.44 ± 0.30	4.45 ± 0.28	4.54 ± 0.29	0.64
Hemoglobin (g/dL)	13.11 ± 1.28	13.40 ± 0.83	13.92 ± 0.89	0.10
Haematocrit (%)	38.10 ± 3.15	38.92 ± 2.16	40.99 ± 2.59 ^a^	0.01
Platelets (10^3^/µL)	272.81 ± 84.04	259.06 ± 29.90	259.07 ± 45.58	0.97
Leukocytes (10^3^/µL)	6.24 ± 1.80	5.36 ± 1.49	6.18 ± 1.74	0.24
Neutrophils (10^3^/µL)	3.03 ± 1.33	2.99 ± 1.01	3.59 ± 1.31	0.33
Lymphocytes (10^3^/µL)	2.19 ± 0.60	1.74 ± 0.64	1.85 ± 0.54	0.09
Monocytes (10^3^/µL)	0.56 ± 0.14	0.41 ± 0.11 ^a^	0.52 ± 0.18	0.02
Eosinophils (10^3^/µL)	0.15 ± 0.09	0.14 ± 0.10	0.18 ± 0.08	0.32
Basophils (10^3^/µL)	0.04 ± 0.01	0.04 ± 0.02	0.05 ± 0.02	0.24
HbA1c (%)	5.22 ± 0.29	5.27 ± 0.28 ^a^	5.75 ± 0.30 ^a^	<0.01

Values are means ± SDs. SD, standard deviation; BMI, body mass index; HbA1c, glycated hemoglobin; *p* < 0.05, significant differences (one-way ANOVA or Kruskal–Wallis test): ^a^ GR-40 or GR-60 vs. GR-20, ^b^ GR-60 vs. GR-40 (*t*-test or Mann–Whitney U test).

**Table 2:** Blood level of markers of carbohydrate and lipid metabolism in the compared groups depending on the number of whole-body cryotherapy sessions.

Variable	Group			Mean ± SD			ANOVA (*p*, η^2^, 1-β)		
		(1) Pre 1 WBC	(2) After 10 WBC	(3) After 20 WBC	(4) After 30 WBC	(5) After 2 Weeks	Group	WBC	Group × WBC
FBG	GR-20	4.78 ± 0.31	4.77 ± 0.29	4.79 ± 0.30	4.59 ± 0.34	4.59 ± 0.31	<0.001	0.032	0.279
(mmol/L)	GR-40	5.02 ± 0.41 ^a^	5.01 ± 0.28	5.06 ± 0.31	4.93 ± 0.31 ^a^	5.04 ± 0.38 ^a^	0.36	0.06	0.05
	GR-60	5.38 ± 0.49 ^a^	5.11 ± 0.48 ^a,b^	5.16 ± 0.40 ^a,b^	5.14 ± 0.45 ^a,b^	5.25 ± 0.59 ^a,b^	0.99	0.74	0.56
T-CHOL	GR-20	4.11 ± 0.72	4.21 ± 0.59	4.54 ± 1.02	4.30 ± 0.69	4.37 ± 0.92	0.005	0.623	0.087
(mmol/L)	GR-40	5.20 ± 0.86 ^a^	5.22 ± 0.79 ^a^	5.21 ± 0.80 ^a^	5.35 ± 0.91 ^a^	5.24 ± 0.89 ^a^	0.22	0.02	0.08
	GR-60	5.16 ± 0.97 ^a^	5.11 ± 1.20 ^a^	5.01 ± 0.91	5.05 ± 0.88 ^a^	4.93 ± 0.93	0.86	0.21	0.75
LDL-C	GR-20	2.11 ± 0.66	2.11 ± 0.59	2.41 ± 0.76	2.24 ± 0.70	2.24 ± 0.80	0.017	0.488	0.071
(mmol/L)	GR-40	2.87 ± 0.63 ^a^	3.00 ± 0.70 ^a^	2.91 ± 0.57	3.02 ± 0.72 ^a^	2.89 ± 0.58 ^a^	0.18	0.02	0.08
	GR-60	2.81 ± 0.98 ^a^	2.76 ± 1.00 ^a^	2.69 ± 0.99	2.72 ± 0.87	2.61 ± 0.90	0.74	0.27	0.77
HDL-C	GR-20	1.64 ± 0.40	1.72 ± 0.36	1.78 ± 0.45	1.76 ± 0.33	1.75 ± 0.36	0.673	0.283	0.223
(mmol/L)	GR-40	1.71 ± 0.35	1.69 ± 0.32	1.73 ± 0.31	1.75 ± 0.31	1.70 ± 0.35	0.02	0.03	0.06
	GR-60	1.66 ± 0.36	1.62 ± 0.36	1.61 ± 0.33	1.65 ± 0.33	1.66 ± 0.28	0.11	0.39	0.60
TG	GR-20	0.74 ± 0.35	0.83 ± 0.35	0.76 ± 0.22	0.69 ± 0.24	0.84 ± 0.31	0.063	0.578	0.506
(mmol/L)	GR-40	1.49 ± 1.98	1.17 ± 0.84	1.17 ± 1.03	1.16 ± 1.02	1.44 ± 1.58	0.12	0.02	0.04
	GR-60	1.52 ± 0.75 ^a^	1.61 ± 0.75 ^a^	1.56 ± 0.70 ^a^	1.49 ± 0.65 ^a^	1.46 ± 0.78	0.54	0.23	0.42
TyG	GR-20	7.94 ± 0.38	7.99 ± 0.40	8.00 ± 0.27	7.77 ± 0.33	7.97 ± 0.35	0.001	0.141	0.427
	GR-40	8.40 ± 0.62 ^a^	8.30 ± 0.53	8.27 ± 0.53	8.25 ± 0.53 ^a^	8.44 ± 0.55 ^a^	0.30	0.04	0.05
	GR-60	8.66 ± 0.58 ^a^	8.69 ± 0.49 ^a,b^	8.66 ± 0.53 ^a,b^	8.62 ± 0.51 ^a,b^	8.60 ± 0.49 ^a^	0.97	0.53	0.46
AIP	GR-20	−0.33 ± 0.26	−0.34 ± 0.25	−0.37 ± 0.20	−0.42 ± 0.19	−0.33 ± 0.23	0.014	0.137	0.421
	GR-40	−0.17 ± 0.34	−0.22 ± 0.30	−0.24 ± 0.31	−0.25 ± 0.28 ^a^	−0.16 ± 0.31	0.18	0.04	0.05
	GR-60	−0.08 ± 0.27 ^a^	−0.04 ± 0.26 ^a^	−0.05 ± 0.26 ^a^	−0.08 ± 0.24 ^a^	−0.10 ± 0.24 ^a^	0.76	0.53	0.47

SD: standard deviation; WBC: whole-body cryotherapy; FBG: fasting blood glucose; T-CHOL: total cholesterol; LDL-C: LDL cholesterol; HDL-C: HDL cholesterol; TG: triglyceride; TyG: triglyceride glucose index; AIP: atherogenic index of plasma; η^2^: partial eta squared—effect sizes for two-way repeated measures ANOVA analysis (0.010–0.059 = small, 0.060–0.139 = medium, ≥0.14 = large effect); 1-β: power of the statistical test; ^a^ and ^b^: statistically significant differences (Bonferroni post hoc test *p* < 0.05), ^a^ GR-60 compared to GR-20 or to GR-40, ^b^ GR-60 compared to GR-40; (1): before 1 WBC—on the day of the first WBC procedure (day 1); (2): after 10 WBC—in the morning, the next day after 10 WBC procedures (day 13); (3): after 20 WBC—in the morning, the next day after 20 WBC procedures (day 27); (4): after 30 WBC—in the morning, the next day after 30 WBC procedures (day 41); (5): after 2 weeks—in the morning, two weeks after the 30 WBC procedure (day 55).

**Table 3:** Level of selected hormone concentrations and insulin-resistance markers in the compared groups depending on the number of whole-body cryotherapy sessions.

Variable	Group			Mean ± SD			ANOVA (*p*, η^2^, 1-β)		
		(1) Pre 1 WBC	(2) After 10 WBC	(3) After 20 WBC	(4) After 30 WBC	(5) After 2 Weeks	Group	WBC	Group × WBC
METRNL	GR-20	0.56 ± 0.61	0.51 ± 0.46	0.52 ± 0.53	0.48 ± 0.43	0.55 ± 0.58	0.260	0.229	0.582
(ng/mL)	GR-40	0.46 ± 0.57	0.46 ± 0.62	0.47 ± 0.66	0.45 ± 0.53	0.45 ± 0.61	0.60	0.03	0.04
	GR-60	0.26 ± 0.08	0.26 ± 0.08	0.26 ± 0.07	0.24 ± 0.07	0.25 ± 0.09	0.28	0.44	0.38
Irisin	GR-20	10.08 ± 6.08	10.49 ± 6.59	10.30 ± 5.28	9.11 ± 4.32	9.57 ± 4.77	0.872	0.927	0.001
(μg/mL)	GR-40	10.50 ± 5.18	10.74 ± 6.64	10.19 ± 5.92	11.20 ± 6.46	8.84 ± 4.57	0.01	0.01	0.14
	GR-60	10.03 ± 5.61	10.36 ± 6.32	10.39 ± 5.35	11.72 ± 6.59	12.64 ± 7.13	0.07	0.10	0.97
Asprosin	GR-20	22.99 ± 20.18	23.57 ± 21.95	24.78 ± 23.61	26.97 ± 20.27	29.45 ± 21.37	0.651	0.395	0.326
(ng/mL)	GR-40	22.15 ± 22.15	22.20 ± 20.86	25.16 ± 21.75	21.76 ± 21.10	21.63 ± 20.03	0.02	0.02	0.05
	GR-60	25.54 ± 39.65	15.06 ± 18.26	25.54 ± 40.50	15.90 ± 21.37	14.80 ± 19.60	0.12	0.32	0.53
Insulin	GR-20	9.59 ± 9.88	8.51 ± 4.41	7.41 ± 4.45	6.35 ± 3.85	6.53 ± 3.25	0.203	0.250	0.045
(μIU/mL)	GR-40	8.60 ± 5.32	6.56 ± 4.16	8.60 ± 6.82	8.70 ± 5.10	8.12 ± 4.94	0.08	0.03	0.10
	GR-60	12.63 ± 6.87 ^a,b^	10.53 ± 5.62	8.40 ± 4.16	9.27 ± 4.02	9.70 ± 4.61	0.33	0.42	0.82
Adiponectin	GR-20	14.12 ± 7.15	12.92 ± 6.15	13.05 ± 5.66	13.22 ± 6.02	13.52 ± 7.32	0.290	0.610	0.997
(μg/mL)	GR-40	11.25 ± 5.26	10.85 ± 4.92	10.22 ± 3.70	11.44 ± 4.88	10.48 ± 4.36	0.05	0.01	0.01
	GR-60	12.80 ± 7.42	12.35 ± 7.88	11.48 ± 6.18	13.34 ± 5.70	11.87 ± 5.75	0.26	0.22	0.09
Leptin	GR-20	13.24 ± 9.93	13.66 ± 11.24	14.43 ± 7.93	13.22 ± 8.60	14.53 ± 9.94	0.061	0.696	0.751
(ng/mL)	GR-40	15.73 ± 7.68	15.54 ± 7.98	15.39 ± 7.04	16.84 ± 7.28	15.69 ± 7.47	0.12	0.01	0.03
	GR-60	21.33 ± 8.08 ^a^	20.58 ± 10.64	20.01 ± 8.14	20.83 ± 9.56 ^a^	22.55 ± 12.89 ^a^	0.55	0.18	0.29
Lept/Adipo	GR-20	1.71 ± 1.15	1.76 ± 1.20	1.80 ± 1.13	1.73 ± 1.31	1.86 ± 1.47	0.007	0.647	0.913
	GR-40	1.33 ± 0.98	1.29 ± 0.91	1.37 ± 1.22	1.25 ± 0.72	1.45 ± 0.79	0.20	0.01	0.02
	GR-60	3.86 ± 6.13 ^a,b^	2.70 ± 3.17	3.04 ± 3.68	2.42 ± 3.52	3.87 ± 4.60 ^a,b^	0.83	0.20	0.19
HOMA-IR	GR-20	2.03 ± 1.99	1.83 ± 0.98	1.80 ± 1.52	1.32 ± 0.82	1.34 ± 0.71	0.127	0.258	0.039
	GR-40	1.95 ± 1.28	1.47 ± 0.96	1.96 ± 1.66	1.92 ± 1.20	1.81 ± 1.11	0.10	0.03	0.10
	GR-60	3.05 ± 1.75 ^a,b^	2.35 ± 1.31	1.92 ± 0.96	2.15 ± 1.01	2.31 ± 1.15 ^a^	0.42	0.41	0.83

SD: standard deviation; WBC: whole-body cryotherapy; METRNL: meteorin-like hormone; Lept/Adipo: leptin-to-adiponectin concentration ratio; HOMA-IR: homeostasis model assessment of insulin resistance; η^2^: partial eta squared—effect sizes for two-way repeated measures ANOVA analysis (0.010–0.059 = small, 0.060–0.139 = medium, ≥0.14 = large effect); 1-β: power of the statistical test; ^a^ and ^b^: statistically significant differences (Bonferroni post hoc test *p* < 0.05), ^a^ GR-60 compared to GR-20 or to GR-40, ^b^ GR-60 compared to GR-40; (1): before 1 WBC—on the day of the first WBC procedure (day 1); (2): after 10 WBC—in the morning, the next day after 10 WBC procedures (day 13); (3): after 20 WBC—in the morning, the next day after 20 WBC procedures (day 27); (4): after 30 WBC—in the morning, the next day after 30 WBC procedures (day 41); (5): after 2 weeks—in the morning, two weeks after the 30 WBC procedure (day 55).

## Data Availability

The data presented in this study are partially available in the RODBUK Cracow Open Research Data Repository (https://doi.org/10.58145/AKF/V6T2YT) and on request from the corresponding author. Some data are not publicly available due to restrictions relating to the protection of participants’ privacy.

## Supplementary Materials

The following supporting information can be downloaded at: https://www.mdpi.com/article/10.3390/ijms27136019/s1.

## Abbreviations

The following abbreviations are used in this manuscript:

AIP	Atherogenic index of plasma
ANOVA	Analysis of variance
BAT	Brown adipose tissue
iBAT	Interscapular brown adipose tissue
BMI	Body mass index
CI	95% confidence intervals
CRP	C-reactive proteins
CV	Coefficient of variation
ERα	Estrogen receptor α
ERβ	Estrogen receptor β
ES	Effect size
FBG	Fasting blood glucose
FP	Follicular phase
GR-20	Group of women aged 20–30
GR-40	Group of women aged 40–50
GR-60	Group of women aged 60–70
HbA1c	Glycated hemoglobin
HDL-C	High-density lipoprotein cholesterol
HOMA-IR	Homeostasis model assessment of insulin resistance
IL	Interleukin
IPAQ	International Physical Activity Questionnaire
LDL-C	Low-density lipoprotein cholesterol
Lept/Adipo	Leptin to adiponectin concentrations ratio
LP	Luteal phase
MCID	Minimal clinically important difference
METRNL	Meteorin-like hormone
mRNA	Messenger RNA
NS	Statistically insignificant difference (*p* > 0.05)
T-CHOL	Total cholesterol
TG	Triglycerides
TNF-α	Tumor necrosis factor α
TyG	Triglyceride–glucose index
WAT	White adipose tissue
eWAT	Epididymal white adipose tissue
sWAT	Subcutaneous white adipose tissue
WBC	Whole-body cryotherapy
WHR	Waist–hip ratio
1-β	Power of statistical test
η^2^	Partial eta squared
*p*	*p*-value
*p* < 0.05	Statistically significant differences
r	Correlation coefficients
α	Significance level (α = 0.05)
