# Supplementary material for: Circulating METRNL, Adipocytokines, and Insulin-Resistance Markers After Repeated Whole-Body Cryotherapy in Women of Different Ages
Source: Int J Mol Sci. 2026 Jul 4;27(13):6019. doi: 10.3390/ijms27136019 (PMC13362472; doi:10.3390/ijms27136019)
Supplement: Supplementary file 1 [file ijms-27-06019-s001.zip › ijms-4287350-supplementary.pdf]

Table S1. The effect of whole-body cryotherapy on changes in the concentration of carbohydrate-lipid metabolism markers in blood in women depending on age and number of treatments.

Variable	Group	Mean (95% CI)				ES (Cohen's d)			
		Δ2-1	Δ3-1	Δ4-1	Δ5-1	Δ2-1	Δ3-1	Δ4-1	Δ5-1
**FBG (mmol/L)**	**GR-20**	0.00 (-0.22-0.21)	0.02 (-0.16-0.20)	-0.18(-0.37-0.01)	-0.20 (-0.41-0.01)	<0.20	<0.20	0.58	0.61
	**GR-40**	-0.01 (-0.20-0.19)	0.04 (-0.19-0.27)	-0.08 (-0.28-0.11)	-0.02 (-0.22-0.19)	<0.20	<0.20	0.23	<0.20
	**GR-60**	-0.27 (-0.47- (-0.07))	-0.22 (-0.41- (-0.03))	-0.23 (-0.45- (-0.02))	-0.13 (-0.42- 0.16)	0.56	0.49	0.51	0.24
**T-CHOL (mmol/L)**	**GR-20**	0.10 (-0.11-0.30)	0.43 (0.13-0.74)	0.20 (0.02-0.37)	0.25 (-0.06-0.57)	<0.20	0.49	0.27	0.32
	**GR-40**	0.02 (-0.21-0.24)	0.00 (-0.24-0.25)	0.14 (-0.15-0.43)	0.01 (-0.35-0.36)	<0.20	<0.20	<0.20	<0.20
	**GR-60**	-0.05 (-0.57-0.48)	-0.14 (-0.43-0.14)	-0.11 (-0.36-0.14)	-0.23 (-0.53-0.08)	<0.20	<0.20	<0.20	0.24
**LDL-C (mmol/L)**	**GR-20**	-0.01 (-0.19-0.18)	0.30 (0.09-0.50)	0.12 (-0.01-0.26)	0.14 (-0.10-0.38)	<0.20	0.42	<0.20	<0.20
	**GR-40**	0.08 (-0.12-0.27)	0.04 (-0.19-0.27)	0.15 (-0.13-0.43)	0.00 (-0.32-0.31)	0.20	<0.20	0.22	<0.20
	**GR-60**	-0.05 (-0.43-0.32)	-0.12 (-0.31-0.08)	-0.09 (-0.31-0.14)	-0.20 (-0.44-0.04)	<0.20	<0.20	<0.20	0.21
**HDL-C (mmol/L)**	**GR-20**	0.08 (-0.02-0.18)	0.14 (0.01-0.28)	0.12 (0.01-0.23)	0.09 (-0.04-0.22)	0.21	0.33	0.33	0.29
	**GR-40**	-0.02 (-0.08-0.03)	0.01 (-0.05-0.08)	0.04 (-0.06-0.13)	0.00 (-0.11-0.11)	<0.20	<0.20	<0.20	<0.20
	**GR-60**	-0.03 (-0.08-0.01)	-0.04 (-0.12-0.03)	-0.01 (-0.10-0.08)	0.00 (-0.09-0.09)	<0.20	<0.20	<0.20	<0.20
**TG (mmol/L)**	**GR-20**	0.09 (-0.09-0.27)	0.02 (-0.13-0.17)	-0.06 (-0.21-0.10)	0.09 (-0.09-0.27)	0.26	<0.20	<0.20	0.30
	**GR-40**	-0.32 (-0.97-0.33)	-0.32 (-0.84-0.20)	-0.33 (-0.84-0.18)	-0.09 (-0.34-0.17)	0.21	0.20	0.21	<0.20
	**GR-60**	0.09 (-0.33-0.51)	0.03 (-0.18-0.25)	-0.03 (-0.37-0.30)	-0.07 (-0.60-0.46)	<0.20	<0.20	<0.20	<0.20
**TyG**	**GR-20**	0.05 (-0.16-0.27)	0.06 (-0.14-0.25)	-0.17 (-0.36-0.01)	0.01 (-0.20-0.22)	<0.20	<0.20	0.48	<0.20
	**GR-40**	-0.11 (-0.31-0.10)	-0.13 (-0.34-0.07)	-0.15 (-0.31-0.00)	0.01 (-0.11-0.12)	<0.20	0.23	0.26	<0.20
	**GR-60**	0.03 (-0.19-0.24)	-0.01 (-0.16-0.14)	-0.04 (-0.26-0.18)	-0.06 (-0.37-0.24)	<0.20	<0.20	<0.20	<0.20
**AIP**	**GR-20**	0.00 (-0.11-0.10)	-0.04 (-0.13-0.05)	-0.09 (-0.18- (-0.01))	-0.01 (-0.11-0.10)	<0.20	<0.20	0.40	<0.20
	**GR-40**	-0.04 (-0.13-0.05)	-0.07 (-0.15-0.02)	-0.07 (-0.14-0.00)	0.00 (-0.05-0.06)	<0.20	0.22	0.26	<0.20
	**GR-60**	0.04 (-0.05-0.14)	0.03 (-0.03-0.08)	0.00 (-0.09-0.09)	-0.02 (-0.16-0.13)	<0.20	<0.20	<0.20	<0.20

CI: confidence interval; WBC: whole-body cryotherapy, Δ2-1: difference after 10 WBC, compared to pre 1 WBC, Δ3-1: difference after 20 WBC compared to pre 1 WBC, Δ4-1: difference after 30 WBC, compared to pre 1 WBC, Δ5-1: difference 2 weeks after 30 WBC compared to pre 1 WBC; FBG: fasting blood glucose, T-CHOL: total cholesterol, LDL-C: LDL-cholesterol, HDL-C: HDL-cholesterol, TG: triglycerides, TyG: triglyceride glucose index, AIP: atherogenic index of plasma; ES: effect size between baseline and post WBC data - Cohen's d (<0.20 none, 0.20 small, 0.50 medium, 0.80 large effect), values are reported if d ≥ 0.20.

Table S2. The effect of whole-body cryotherapy on changes in the selected hormones blood concentration and insulin resistance index in women depending on age and number of treatments.

Variable	Group	Mean (95% CI)				ES (Cohen's d)			
		Δ2-1	Δ3-1	Δ4-1	Δ5-1	Δ2-1	Δ3-1	Δ4-1	Δ5-1
**METRNL (ng/mL)**	**GR-20**	-0.05(-0.14-0.05)	-0.04 (-0.11-0.02)	-0.07 (-0.18-0.03)	-0.04 (-0.09-0.01)	<0.20	<0.20	<0.20	<0.20
	**GR-40**	0.00 (-0.03-0.04)	0.01 (-0.04-0.06)	0.00 (-0.06-0.05)	-0.02 (-0.06-0.02)	<0.20	<0.20	<0.20	<0.20
	**GR-60**	0.00 (-0.04-0.04)	0.01 (-0.03-0.04)	-0.02 (-0.06-0.01)	-0.01 (-0.05-0.03)	<0.20	<0.20	<0.20	<0.20
**Irisin (µg/mL)**	**GR-20**	0.41 (-1.21-2.03)	0.23 (-1.34-1.79)	-0.96 (-2.92-0.99)	-0.87 (-2.51-0.76)	<0.20	<0.20	<0.20	<0.20
	**GR-40**	0.24 (-2.45-2.93)	-0.31 (-2.10-1.49)	0.70 (-1.25-2.65)	-2.01 (-3.38-(-0.63))	<0.20	<0.20	<0.20	0.34
	**GR-60**	0.34 (-1.38-2.05)	0.37 (-0.91-1.64)	1.69 (-0.67-4.06)	2.62 (-0.34-5.57)	<0.20	<0.20	0.28	0.41
**Asprosin (ng/mL)**	**GR-20**	0.58 (-3.57-4.72)	1.78 (-4.77-8.34)	3.97 (0.19-7.76)	5.25 (-2.00-12.51)	<0.20	<0.20	0.20	0.31
	**GR-40**	0.06 (-4.06-4.17)	3.01 (-4.23-10.26)	-0.39 (-4.59-3.80)	-1.70 (-8.10-4.70)	<0.20	<0.20	<0.20	<0.20
	**GR-60**	-10.48 (-31.87-10.92)	0.00 (-1.09-1.09)	-9.64 (-30.99-11.71)	-10.74 (-32.12-10.63)	0.34	<0.20	0.30	0.34
**Insulin (µIU/mL)**	**GR-20**	-1.08 (-6.75-4.58)	-2.00 (-8.59-3.92)	-3.23 (-8.59-2.12)	-0.51 (-2.54-1.51)	<0.20	0.28	0.43	0.40
	**GR-40**	-1.77 (-4.47-0.93)	-0.13 (-3.44-3.18)	0.33 (-2.76-3.42)	-0.91 (-2.69-0.87)	0.43	<0.20	<0.20	<0.20
	**GR-60**	-2.36 (-4.92-0.20)	-4.23 (-7.39-(-1.07))‡	-3.36 (-6.65-(-0.07))	-2.93 (-5.96-0.09)	0.33	0.74	0.60	0.50
**ADIPO (µg/mL)**	**GR-20**	-1.20 (-3.93-1.53)	-1.07 (-3.42-1.29)	-0.90 (-2.61-0.81)	-0.60 (-4.76-3.57)	<0.20	<0.20	<0.20	<0.20
	**GR-40**	-0.40 (-3.56-2.77)	-1.03 (-2.99-0.92)	0.19 (-2.05-2.43)	-0.77 (-2.73-1.19)	<0.20	0.23	<0.20	<0.20
	**GR-60**	-0.46 (-3.66-2.74)	-1.32 (-7.12-4.47)	0.54 (-1.83-2.91)	-0.93 (-4.24-2.37)	<0.20	<0.20	<0.20	<0.20
**Leptin (ng/mL)**	**GR-20**	0.43 (-2.64-3.49)	1.20 (-1.91-4.30)	-0.01 (-2.09-2.06)	1.30 (-2.00-4.59)	<0.20	<0.20	<0.20	<0.20
	**GR-40**	-0.19 (-2.34-1.95)	-0.34 (-2.82-2.13)	1.10 (-1.37-3.58)	-0.04 (-2.66-2.57)	<0.20	<0.20	<0.20	<0.20
	**GR-60**	-0.76 (-3.78-2.26)	-1.32 (-3.56-0.92)	-1.50 (-2.95-1.94)	1.22 (-2.99-5.42)	<0.20	<0.20	<0.20	<0.20
**Lept/Adipo**	**GR-20**	0.05 (-0.16-0.26)	0.09 (-0.05-0.24)	0.03 (-0.18-0.24)	0.15 (-0.14-0.44)	<0.20	<0.20	<0.20	<0.20
	**GR-40**	-0.03 (-0.27-0.20)	0.05 (-0.29-0.38)	-0.08 (-0.36-0.20)	0.12 (-0.20-0.44)	<0.20	<0.20	<0.20	<0.20
	**GR-60**	-1.16 (-5.05-2.74)	-0.81 (-2.98-1.36)	-1.43 (-5.44-2.57)	0.02 (-3.76-3.79)	0.24	<0.20	0.29	<0.20
**HOMA-IR**	**GR-20**	-0.20 (-1.35-0.96)	-0.26 (-1.69-1.17)	-0.71 (-1.77-0.36)	-0.17 (-0.62-0.28)	<0.20	<0.20	0.47	0.45
	**GR-40**	-0.40 (-1.06-0.26)	-0.01 (-0.89-0.86)	0.03 (-0.72-0.78)	-0.23 (-0.64-0.17)	0.42	<0.20	<0.20	<0.20
	**GR-60**	-0.75 (-1.47-(-0.03))	-1.14 (-1.95-(-0.32))‡	-0.90 (-1.76-(-0.05))	-0.74 (-1.52-0.03)	0.45	0.80	0.63	0.50

CI: confidence interval; WBC: whole-body cryotherapy, Δ2-1: difference after 10 WBC, compared to pre 1 WBC, Δ3-1: difference after 20 WBC compared to pre 1 WBC, Δ4-1: difference after 30 WBC, compared to pre 1 WBC, Δ5-1: difference 2 weeks after 30 WBC compared to pre 1 WBC; METRNL: meteorin-like hormone, ADIPO: adiponectin, Lept/Adipo: leptin

to adiponectin concentrations ratio, HOMA-IR: homeostasis model assessment of insulin resistance; ‡: statistically significant difference (Bonferroni post-hoc test $p<0.05$); ES: effect size between baseline and post WBC data - Cohen's d (<0.20 none, 0.20 small, 0.50 medium, 0.80 large effect), values are reported if $d \geq 0.20$.
